# Pressure Injury Prevention in COVID-19 Patients With Acute Respiratory Distress Syndrome

**DOI:** 10.3389/fmed.2020.558696

**Published:** 2021-01-22

**Authors:** Victoria Team, Lydia Team, Angela Jones, Helena Teede, Carolina D. Weller

**Affiliations:** ^1^Monash Nursing and Midwifery, Faculty of Medicine, Nursing and Health Sciences, Monash University, Melbourne, VIC, Australia; ^2^Monash Partners Academic Health Science Centre, Clayton, VIC, Australia; ^3^Monash Health, Clayton, VIC, Australia; ^4^School of Nursing and Midwifery, Deakin University, Melbourne, VIC, Australia

**Keywords:** acute respiratory distress syndrome, COVID-19, guidelines, intensive care, pressure injury, pressure points, prone positioning, ventilation

## Abstract

Coronavirus disease 2019 (COVID-19), which is caused by severe acute respiratory syndrome coronavirus 2 (SARS-CoV-2), was identified in China in December 2019 and became a pandemic in a short period of time. While most infected people might have mild symptoms, older people and people with chronic illnesses may develop acute respiratory distress syndrome (ARDS). Patients with ARDS with worsening hypoxemia require prone positioning to improve the respiratory mechanics and oxygenation. Intubated patients may stay in a prone position up to 12–16 h, increasing the risk of pressure injury (PI). Frequent skin inspections and PI risk assessment in COVID-19 patients will be challenging due to hospital infection control measures aimed to reduce the risk for health professionals. In this perspective article, we summarize the best practice recommendations for prevention of PI in SARS-CoV-2-infected ARDS patients in prone positioning. Prior to positioning patients in prone position, the main recommendations are to (1) conduct a skin assessment, (2) use pressure redistribution devices, (3) select an appropriate mattress or an overlay, (4) ensure that the endotracheal tube securing device is removed and the endotracheal tube is secured with tapes, (5) use a liquid film-forming protective dressing, and (6) lubricate the eyes and tape them closed. Once a patient is in prone position, it is recommended to (1) use the swimmer's position, (2) reposition the patient every 2 h, and (3) keep the skin clean. When the patient is repositioned to supine position, healthcare professionals are advised to (1) assess the pressure points and (2) promote early mobilization.

## Introduction

Coronavirus disease 2019 (COVID-19), which is caused by severe acute respiratory syndrome coronavirus 2 (SARS-CoV-2), was identified in China in December 2019 and rapidly became a pandemic (1, 2). While most infected people have mild symptoms, older people and people with chronic illnesses may become critically ill and develop viral pneumonia and acute respiratory distress syndrome (ARDS) (3–5), requiring admission to an intensive care unit (ICU) (6). The pathophysiology of SARS-CoV-2 ARDS differs from that of the typical ARDS (7). The pathophysiological mechanism of COVID-19-related ARDS is pulmonary micro-thrombosis (8). The results of lung and skin biopsy of critically ill SARS-CoV-2-infected patients demonstrated generalized thrombotic microvascular injury (7). SARS-CoV-2 infection results in cytokine storm and a local and systemic inflammatory response syndrome leading to macro- and microthrombosis (8, 9). The three factors of Virchow's triad—reduced blood flow, endothelial injury, and hypercoagulability—increase the risk of thrombosis in severe COVID-19 patients (8).

Most ICU-admitted COVID-19 patients need non-invasive ventilation, a non-rebreathing mask, and prone positioning to increase oxygen delivery (10) as well as high-flow nasal oxygen through specialized nasal cannula in negative-pressure rooms (11). Up to 5% of COVID-19 patients with ARDS may require endotracheal intubation (12, 13). In general, patients with ARDS of any etiology with worsening hypoxemia (PaO_2_:FiO_2_ < 100–150 mmHg, FiO_2_ ≥ 0.6, PEEP ≥ 10 cm of water, and tidal volume of 6 ml/kg of predicted body weight) require prone positioning to improve the respiratory mechanics, improve oxygenation, and offload the weight of the heart (14, 15). When applied early (usually 12 to 24 h after the initiation of mechanical ventilation) with other lung-protective strategies and adopted for a prolonged period, the prone position is associated with reduced mortality, particularly in patients with severe hypoxemia (16–18).

Prone positioning was reported in the management of ARDS in critically ill patients with severe acute respiratory syndrome (SARS) (19) and Middle East respiratory syndrome (MERS) (20) coronavirus infections and is used to manage ARDS in COVID-19 patients (12). Initially, it was found to be effective in one small-scale study of 52 critically ill patients with SARS-CoV-2 pneumonia in Wuhan, China (21), with further anecdotal evidence from day-to-day clinical practice in ICU. A recent small-scale study (22) on the use of prone positioning in non-intubated patients with COVID-19 and hypoxemic acute respiratory failure in Turkey, managed outside the ICU, reported that oxygenation increased in only a quarter of patients and was not sustained in half of those after resupination. Self-proning of COVID-19 patients is increasingly used in several countries and has become a standard treatment in the management of ARDS patients with hypoxia (23). With some precautions, prone positioning is used in the management of COVID-19-related ARDS in pregnant women (24). A case report from Japan suggests that, although prone positioning may mitigate hypoxemia, its role in reducing mortality in COVID-19 patients with ARDS is unclear, particularly in patients with a secondary superinfection (25), which is often associated with sepsis, shock, and multiple organ failure (26).

The intubated patients may remain in a prone position up to 16 h per day, alternating with 8 h in supine position (27). Prone positioning increases the risk of developing hospital-acquired pressure injury (HAPI) (16, 28), and this risk is higher when compared to the supine position (29). A Wuhan study reported that the mean hospital stay of COVID-19 patients with pneumonia was 22 days (5); prolonged ICU admission (30) and increased hospital stay (31) are independent risk factors for the development of HAPI.

While the studies reporting HAPI incidence in COVID-19 ARDS patients have not yet been published, individual case reports have reported that patients cared in prone position are at risk of developing multiple severe device-related PI on their face, requiring a consultation and an intervention by plastic surgeons (32). Furthermore, diarrhea is a common gastrointestinal feature in COVID-19 patients (33, 34), and in addition to other risk factors, such as immobility and reduced perfusion, diarrhea may contribute to the development of incontinence-associated dermatitis and a pressure injury in the sacral area (35). This risk is even higher in older patients with COVID-19 ARDS and requires immediate attention (36).

Repositioning and pressure relief are important strategies to reduce the risk (37). However, clinical experience reports the need to involve up to seven people to reposition the intubated patient. Frequent skin inspections and risk assessment in COVID-19 patients could be challenging due to hospital infection control measures aimed to reduce the risk for health professionals working in ICU (11, 38).

In general, there is a global health professional knowledge deficit on PI prevention, with early detection (39–45) and standard preventive interventions recommended in clinical practice guidelines not fully implemented (46) in the context of COVID-19 clinical care. Health professionals may lack awareness of pressure points typical for patients in prone position and may have misconceptions related to the specific equipment required for prone positioning (47). In this perspective article, we summarize the best recommendations for the prevention of PI in SARS-CoV-2-infected ARDS patients in prone position.

## Prone Position: Pressure Injury Prevention

The latest version of *Prevention and Treatment of Pressure Ulcers/Injuries: Clinical Practice Guideline, International Version* (37) acknowledges evidence derived from one low-quality study (29), indicating that prone positioning is associated with a higher incidence of HAPI compared with supine positioning. The reported incidence of HAPI is said to be 5 to 15% as derived from low- and moderate-quality studies (48–50). The main recommendation is to avoid the extended use of prone positioning unless required for the management of a medical condition ((37), p. 126). However, COVID-19 ARDS management requires prone positioning for extended periods of time, and therefore, using appropriate support surfaces and pillows and patient repositioning as soon as feasible are key preventive strategies recommended by the guidelines (37). Facial pillows and chest padding can be used to redistribute pressure. The main pressure points in the prone position are the forehead, chin, cheeks, shoulder (anterior), elbow, chest (breasts), genitalia (particularly male), anterior pelvic bones (iliac crests and ischium), knees (patella), dorsal feet and toes, and nose (if positioned incorrectly), which should be inspected as soon as feasible ((37), p. 139), especially if supplies of personal protective equipment are limited.

According to the guidelines ((37), p. 126), the implementation strategies for HAPI prevention in the prone position include the following:

1. Use of pressure redistribution support surface or positioning devices to offload pressure points on the face and the body,
2. Checking for uneven pressure redistribution, focusing on main pressure points unique to prone position, and positioning of medical devices,
3. Use of additional PI preventive strategies, including prophylactic silicone dressings over the bony prominences and under medical devices,
4. Assessing the face and body areas in the main pressure points at each rotation.

A recent review (51) of PI prevention in non-COVID-19 patients, placed in prone position, reported that the main preventive strategies include (1) conducting a skin assessment before proning and following repositioning to the supine position, (2) keeping the skin clean and moisturized, (3) repositioning to offload pressure points on the face and the body, (4) use of positioning devices, and (5) application of dressings, such as hydrocolloids, transparent film, and silicone, to decrease facial skin breakdown (51). Practical suggestions provided by Wounds International (52) include the need to (1) protect bony prominences on the front of the body prior to prone positioning, (2) lubricate the eyes and tape them closed, (3) select an appropriate mattress or an overlay, (4) ensure that the endotracheal tube securing devices are removed—the endotracheal tube should be secured with tapes with the help of a respiratory therapist, (5) ensure the use of liquid film-forming dressing such as SKIN-PREP to decrease trauma on removal, and (6) place the patient's face in swimmer's position when prone, i.e., turn the face to the side toward a flexed arm and put the other arm behind the patient. The swimmer's position allows movement of the head and the endotracheal tube (and a nasogastric tube) at the same time, which should be done every 2 h.

**Figure 1:**
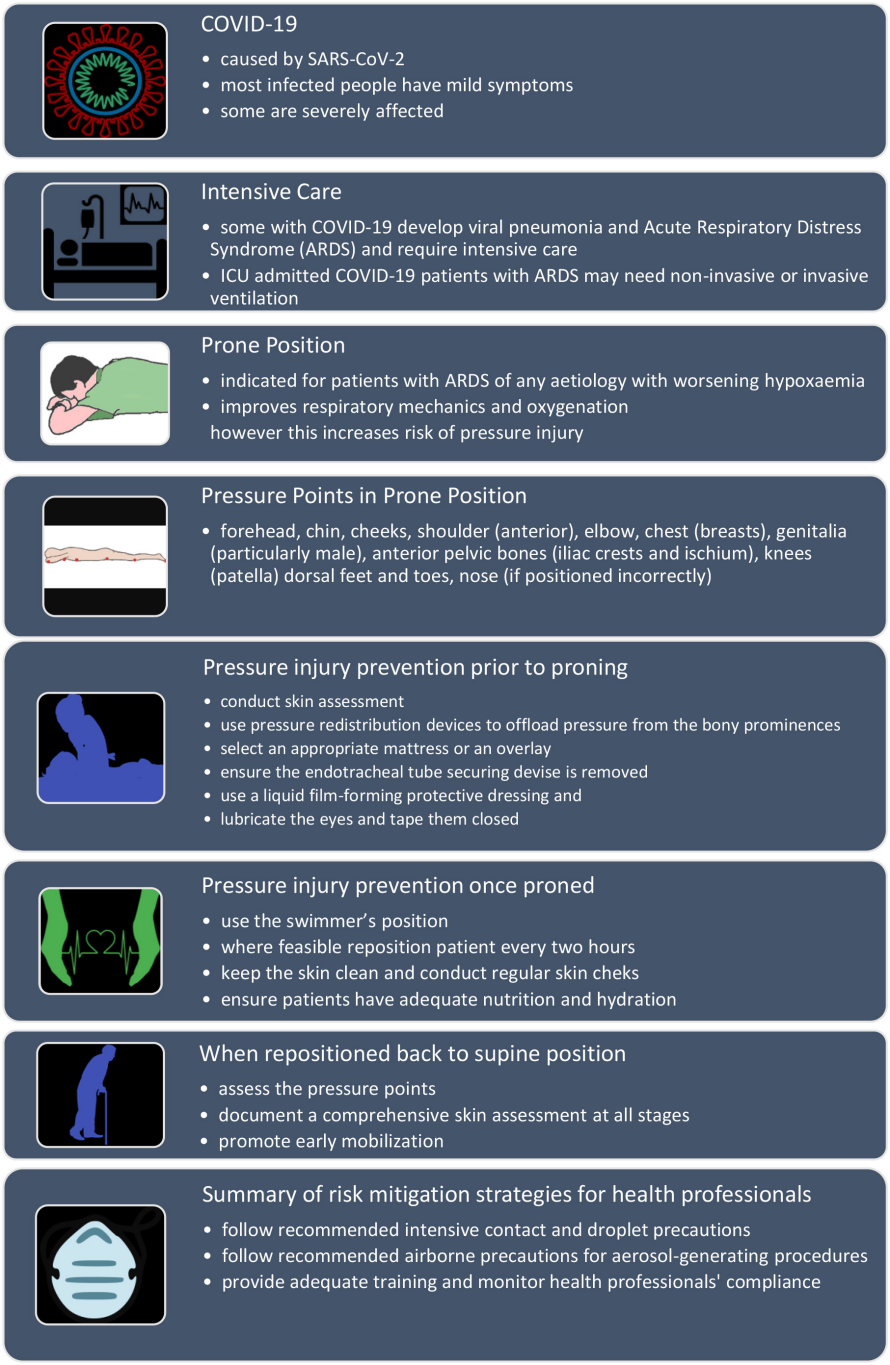
COVID-19. Pressure Injury prevention in ARDS patients in prone position. Infographic.

We have summarized the main points of PI prevention in patients in prone position in the infographic (Figure 1).

Finally, considering the pathophysiology of SARS-CoV-2 in relation to severe thrombosis (7, 8), patient repositioning as well as early mobilization should be prioritized. Specific guidelines with detailed instructions on how to prepare patients in prone position for care and how to reposition in the supine position are available for intensive care units (15, 53).

The patient's position and the duration in prone position need to be well-documented. Repositioning of unconscious patients into a prone position should be conducted by a team of at least four health professionals, following individual hospital policies, standard safety practices (37), and COVID-19 occupational safety guidelines. PI management relies on team work (37), and the assistance of other team members may be required (54), which should be arranged according to the risk mitigation strategies for health professionals (55).

The main routes of occupational SARS-Cov-2 transmission for health professionals include droplets, airborne transmission, especially during invasive respiratory procedures, and contact transmission (26). In addition to standard measures, health professionals should apply contact and droplet precautions and airborne precautions for aerosol-generating procedures to mitigate the risk of SARS-Cov-2 transmission (56). The main risk mitigation strategies for health professionals (Table 1) include the need to be trained in fastidiously applying, wearing, and removing personal protective equipment, which prevents droplets, contact, and airborne transmission; to perform aerosol-generating procedures in a well-ventilated environment, preferably in a negative-pressure room; to allocate a team of healthcare workers to care exclusively for suspected/confirmed cases; and to limit the number of healthcare workers present in the room to the absolute minimum required for the patient's care and support (56).

**Table 1:** COVID-19 patient care: Empiric additional precautions^*^.

**Contact and droplet precautions**	
Personal protective measures	• use a medical mask • wear eye or facial protection (goggles or face shield) • wear a clean, non-sterile, long-sleeved gown • use gloves • remove and dispose of all personal protective equipment appropriately • maintain hand hygiene • use a new set of personal protective equipment when care is given to a different patient • refrain from touching face with potentially contaminated gloved or bare hands
Equipment-related precautions	• use either single-use and disposable or dedicated equipment (e.g., thermometers, stethoscopes, and blood pressure cuffs) • if shared among patients, clean and disinfect equipment between use for each individual patient (e.g., ethyl alcohol 70%) • use designated portable X-ray or other diagnostic equipment
Engineering and environment-related precautions	• place patients in adequately ventilated single rooms (60 L/s per patient) • group patients together when single rooms are not available • ensure patients' beds are placed at least 1 meter apart regardless of the suspected COVID-19 diagnosis • clean and disinfect surfaces with which the patient is in contact
Patient transporting precautions	• avoid moving and transporting patients unless medically necessary • use predetermined transport routes to minimize exposure for staff, other patients and visitors, and have the patient wear a medical mask • ensure that health care workers transporting patients wear appropriate personal protective equipment and perform hand hygiene • notify the area receiving the patient of any necessary precautions as early as possible prior to their arrival
Administrative measures	• where possible, designate a team of health care workers to care exclusively for suspected/confirmed cases to reduce the risk of COVID-19 transmission • limit the number of staff, family members, and visitors who are in contact with suspected/confirmed COVID-19 patients • maintain a record of all people entering a patient's room
**Airborne precautions for aerosol-generating procedures**	
Personal protective measures	• use a particulate respirator approved by country-specific occupational safety and health standard • perform the seal check, if you use a disposable particulate respirator • note that facial hair may prevent a proper respirator fit • use eye protection (i.e., goggles or a face shield) • wear a clean, non-sterile, long-sleeved gown and gloves • wear a waterproof apron, if a gown is not fluid-resistant
Engineering and environment-related precautions	• perform procedures in an adequately ventilated room (“natural ventilation with air flow of minimum 160 L/s per patient or in negative pressure rooms with at least 12 air changes per hour and controlled direction of airflow when using mechanical ventilation”)
Administrative measures	• limit the number of health professionals present in the room to the absolute minimum required for the patient's care and support

* *These precautions were adapted from the World Health Organization Infection prevention and control during health care when COVID-19 is suspected. Interim guidance. 19 March 2020*.

## Discussion

Prone positioning may be effective in the management of SARS-CoV-2 ARDS (10), although this position is associated with an increased risk of HAPI (28). HAPI is a well-known indicator of the quality of care in acute settings (57). Patient influx coupled with a shortage of nursing staff and related caregiver fatigue may influence the quality of care. Preventable PI in acute care can interfere with the patients' recovery, can increase hospital stay, and may contribute to death from PI complications, such us osteomyelitis and sepsis (58). Stages III and IV PI are frequently colonized with methicillin-resistant *Staphylococcus aureus* (59) and multi-resistant Gram-negative bacilli (60), which increase the risk of bacteremia (58) and associated mortality (61). We have discussed the main recommendations for PI prevention in COVID-related ARDS patients in prone position from the latest version of *Prevention and Treatment of Pressure Ulcers/Injuries: Clinical Practice Guideline, International Version* (37) and included practical suggestions from the field. In summary, they include specific recommendations for the preparatory stage, care in prone position, and care after repositioning in supine position.

Prior to positioning patients in prone position, the main recommendations are to (1) conduct a skin assessment, (2) use pressure redistribution devices to offload pressure from bony prominences, (3) select an appropriate mattress or an overlay, (4) ensure that the endotracheal tube securing device is removed and that the endotracheal tube is secured with tapes, (5) use a liquid film-forming protective dressing, and (6) lubricate the eyes and tape them closed.

Once the patient is prone, it is recommended to (1) use the “swimmer's position,” i.e., turn the face on the side toward a flexed arm and put the other arm behind the patient, (2) reposition the patient every 2 h, i.e., turn the patient's face to the left and lift the left arm if their face was positioned to the right and their right hand was extended, and (3) keep the skin clean.

When the patient is repositioned to supine position, health care professionals are advised to (1) assess the pressure points and (2) promote early mobilization.

The management of PIs is costly to health systems (62–67). Studies show that PI prevention is more cost-effective than treatment (68). Prevention of HAPI in COVID-19 patients would help to avoid additional financial burden to an increasingly drained health system (69–71), particularly in countries significantly impacted by the COVID-19 outbreak (72). In order to preserve healthcare resources and to ensure adequate hospital capacity for the management of COVID-19 patients, many countries have deferred elective surgeries (73–77) and extended elective surgery waiting time. When the restrictions on elective surgeries are lifted, a sizable proportion of hospital beds might be occupied by COVID-19 patients requiring HAPI care if the preventive practices were suboptimal, given that patients with HAPIs have longer adjusted length of hospital stay (63). In addition to health system costs, there are extreme human costs associated with PI development (66), which further strengthens the importance of prevention of HAPIs in COVID-19 patients. Finally, the predicted second wave of COVID-19 cases (78), the lack of evidence on acquired immunity after COVID-19, and the risk of potential re-infection (79) in the absence of a COVID-19 vaccine (80) may result in increased hospital admissions, highlighting the need to speed up quality improvement in this field.

## Data Availability Statement

The original contributions presented in the study are included in the article/supplementary material, further inquiries can be directed to the corresponding author/s.

## Author Contributions

VT and LT conducted the literature search and drafted the manuscript with support and guidance from CW, AJ, and HT. VT designed the infographic. All the authors critically reviewed and contributed to the individual parts of the manuscript and approved the final version.

## Conflict of Interest

The authors declare that the research was conducted in the absence of any commercial or financial relationships that could be construed as a potential conflict of interest.
